# Ageing of Red Wine (*cv*. Negroamaro) in Mediterranean Areas: Impact of Different Barrels and Apulian Traditional Amphorae on Phenolic Indices, Volatile Composition and Sensory Analysis

**DOI:** 10.3390/foods14040650

**Published:** 2025-02-14

**Authors:** Ilaria Prezioso, Giuseppe Corcione, Chiara Digiorgio, Gabriele Fioschi, Vito Michele Paradiso

**Affiliations:** Department of Biological and Environmental Sciences and Technologies, University of Salento, S.P. 6, 73100 Lecce, Italy; ilaria.prezioso@unisalento.it (I.P.); giuseppe.corcione@unisalento.it (G.C.); chiara.digiorgio@unisalento.it (C.D.); gabriele.fioschi@unisalento.it (G.F.)

**Keywords:** ozza, 3-methyl-2,4-nonanedione, β-damascenone, furaneol, free-choice profiling, terracotta, earthenware

## Abstract

This study investigated the impact of different ageing containers on the volatile composition and quality of Negroamaro wine, a key variety from Apulia, Italy. Seven vessel types were evaluated: traditional Apulian amphorae (ozza), five types of oak barrels (American oak, French oak, European oak, a French + European oak and a multi-wood mix) and glass bottles as the control. The impact of the vessels was evaluated after 6 months of ageing through the characterization of phenolic, volatile and sensory profiles. Amphorae allowed a specific evolution of the wine’s primary aromas, such as fruity and floral notes, while enhancing volatile compounds like furaneol, which contributed to caramel and red fruit nuances, and also 3-methyl-2,4-nonanedione, a key compound related to anise, plum and premature ageing, depending on its concentration. This container also demonstrated effectiveness in stabilizing anthocyanin–tannin complexes, supporting color stabilization. Oak barrels allowed different outcomes to be obtained in terms of color stabilization, volatile profile, aroma and astringency. French oak exhibited the highest phenolic and tannin levels, enhancing anthocyanin stabilization and color intensity. European oak followed closely, while American oak excelled in color stabilization, with tannins less reactive to polymers. Mixed wood barrels showed lower phenolic extraction and the best astringency evolution.

## 1. Introduction

Ageing is a winemaking step that strongly influences wine evolution, and involves a series of chemical and physical reactions that significantly modify wine composition and affect its stability [1,2,3]. It is possible to choose among several ageing materials that have a different impact both on chemical and sensory characteristics of wines. The choice depends mostly on costs, varietal characteristics and oenological goals [3,4]. 

Stainless steel is an inert material that releases negligible amounts of substances into wine. It is used widely and appreciated, especially in large-scale production, as it can process high volumes and ensure high levels of sanitization, pressure resistance, temperature control and easy cleaning and maintenance; therefore, the resulting product can be standardized [5,6].

Wood is widely used in winemaking as consumers appreciate the spicy, toasted and smoked notes that it confers to the wine [1]. Its interaction with wine is related to the porosity degree and the oxygen transmission rate (OTR) of the barrel staves, which allow micro-oxygenation; by introducing controlled amounts of oxygen into the wine, wood promotes color stabilization and improves aroma and texture [7]. This allows an extensive exchange of substances, including hydrolysable tannins and volatile compounds [8,9], in different amounts based on species, geographical origin, toasting level, age of the barrel, time of contact, etc. [3]. However, barrels have some drawbacks, such as a short shelf life, high production time, high production costs and difficulties in sanitization [1].

Finding new ageing materials is important for preserving wine quality, differentiating production and increasing the sustainability of winemaking. Therefore, increasing attention is being paid to earthenware. It is a material with varying degrees of porosity, mainly based on the raw materials and the production technique, and with a suitable coating, it provide several advantages: it enables temperature control and can impart micro-oxygenation benefits without conferring exogenous volatile and phenolic compounds that may cover wine’s varietal characteristics [10,11].

The employment of earthenware to produce ageing containers such as amphorae or Georgian qvevri also has a historical relevance, as it was the first material used to produce wine containers [12].Therefore, its revival is increasingly attracting attention in the wine world [12]. Also, in Apulia, a southern Italian region, earthenware vessels were traditionally produced and called ‘’capasoni’’ or ‘’ozze’’ and they are still used in some areas during production and to stock and transport wine and other foods [10,13,14].

Negroamaro is a variety of *Vitis vinifera* cultivated in Salento (the southern area of Apulia), whose wines have become important for the Italian wine market [15]. Negroamaro is a non-aromatic variety; therefore, it is important to preserve its nature, enhance the stability of its aroma and color and improve its sensorial complexity. In particular, the preservation of primary volatile compounds can be a relevant goal as they are at risk of disappearing in grapes and, consequently, in wines due to global warming [16]. Also, finetuning the sensory impact of wine tannins has become a primary goal in a climate change scenario, since the phenolic maturity of grapes, and particularly of grape seeds, is decoupling with technological maturity and the resulting wines can be characterized by undesirable astringency subqualities and/or bitterness [17,18]. Finally, color appears to be another critical issue for red wines produced in warm climates due to reduced anthocyanin accumulation, extraction and stability [19].

With this view in mind, the effect of five different types of barrels and a traditional Apulian amphora (called ozza) was evaluated on a Negroamaro wine. Color and phenolic indices, volatile profile and sensory characteristics were analyzed and compared after 6 months of ageing.

## 2. Materials and Methods

### 2.1. Ageing Experiment

The Negroamaro wine used for the trial (vintage 2021) was provided by the winery Vecchia Torre (Leverano, Italy). The wine had 14.1% ethanol, pH 3.6, total acidity 6.7 gL^−1^ tartaric acid and total SO_2_ 126 mg L^−1^. Wine was distributed into five types of 225 L barrels (American oak, French oak, European oak, mixed, French + European oak), as described in Table 1, provided by Toneleria Nacional Italy s.r.l. (Barberino Val d’Elsa, Florence, Italy), and traditional Apulian amphorae (ozze) with a capacity of 150–200 L (Figure S1). Amphorae were obtained from a historic smelter in San Pietro in Lama (Lecce, Apulia, Italy), and were produced according to the traditional manufacturing process: the pieces were modelled manually, cooked in an oven at 1000–1100 °C, assembled and finally glazed internally and externally. After filling, amphorae were closed with ceramic dishes and sealed with lime [20]. Three barrels per type and two amphorae were used for the experiment. Ageing was carried out for six months in an underground cellar at a temperature ranging between 16 and 18 °C and a relative humidity approximately of 80%. After fermentation, the same wine was bottled in dark glass 0.75 L bottles, closed with a cork cap, stored and used as a control kept in the same cellar conditions.

### 2.2. Chemical Analysis

Chemical analyses of samples were conducted using an FTIR Winescan FT 120 (Foss, Hillerød, Danmark) analyzer. Color parameters were determined with the modified Somers method [21]. Total phenols were analyzed by Folin–Ciocâlteu assay [22], free and total anthocyanins using the Di Stefano and Cravero method [23], total tannins with the Ribereau–Gayon and Stonestreet method [24] and the tannin–anthocyanin complex with the Glories method [25]. Astringency was evaluated with a methylcellulose precipitation assay (MCPT) [21].

### 2.3. Analysis of Volatile Compounds

Volatile compounds were analyzed and quantified by solid phase microextraction–gas chromatography/mass spectrometry (SPME-GC/MS) according to Lukić and Horvat (2017) [26]. A sample chromatogram and the table of retention times of volatile compounds are reported in the Supplementary Materials (Figure S2 and Table S1, respectively). Odor activity values (OAV) were determined for volatile compounds as the ratios between the measured concentration and the odor threshold reported in the literature [27,28,29,30,31,32,33,34,35,36,37,38,39,40,41,42,43,44,45,46,47,48,49,50,51] (Table S1).

### 2.4. Sensory Analysis

Free choice profiling was carried out to characterize the wines [22]: judges freely assessed and described the characteristics of each wine using a free vocabulary of sensory descriptors, with the only request being to avoid the use of hedonic descriptors [52,53]. A panel composed of 8 judges (5 males, 3 females; age 23–48), winemakers and professionals with experience in wine tasting and knowledge of Apulian cultivars participated in wine evaluation sessions. One training session was carried out to familiarize participants with the sensory methodology, and further training was not required as the method was based on free description [22,53,54]. The experimental replicates were independently analyzed. Samples were coded and presented in random order in glasses complying with the requirements of the ISO 3591 (2) standard [55], at serving temperature (17 ± 2 °C). Panelists individually evaluated each wine in an open-plan sensory facility with a forced 1 min break between each wine, with water and plain crackers available to cleanse the palate. Two sensorial sessions were carried out. In each session, two batches of 4 or 5 samples were analyzed, with an interval between batches for rinsing and de-fatiguing the mouth [56].

The textual data were pre-processed according to Prezioso et al. [22], removing mistakes, eliminating connectors and auxiliary terms, lemmatizing, regrouping synonyms and managing ambiguous words (polysemy and homographs). The frequency of occurrence of sensory descriptors was acquired and submitted to statistical analysis.

### 2.5. Statistical Analysis

One way and two-way analysis of variance (ANOVA), Tukey’s post hoc test, heatmap with cluster analysis, Fisher’s LSD test and principal component analysis (PCA) were carried out with Origin Pro 2022 (OriginLab, Northampton, MA, USA). Correspondence analysis (minimum term frequency = 3) and co-occurrence network (minimum term frequency = 3, filter edges = Jaccard, top 40 edges) were conducted on the results of sensory analysis using the KH coder software, version 3 (http://khcoder.net/en/) [57].

Principal components analysis (PCA), partial least squares discriminant analysis (PLS-DA, with 5-fold cross-validation) and heatmap clustering with Euclidean distance (Ward method) were performed using the MetaboAnalyst6.0 platform (www.metaboanalyst.ca). Data autoscaling was applied as a pretreatment.

## 3. Results

### 3.1. Phenols

Table 2 shows the phenolic indices, MCPT assay and color parameters of samples at initial time (T-0) and after 6 months of ageing in different vessels. The phenolic and color indices evidenced different interactions between the ageing materials and the wine. As expected, contact with wood led to a release of phenolic compounds, mostly ellagitannins [58], as shown by the higher levels of total phenols and total tannins compared to the others. Among woods, French oak held the highest values of total phenols (86.30 a.u.), followed by European oak, French + European oak and finally American oak. A greater concentration of total phenols in French oak barrels, compared to American oak ones, was also reported by [59,60]. As a consequence, an increase in tannin value occurred during ageing, reaching a maximum in American and French oak. The mixed barrels demonstrated a lower total phenol concentration compared to other woods, maybe due to a lower release of ellagitannins, which was also confirmed by a low total tannin value (3.27 g L^−1^), which could also explain the low astringent perception reported during sensorial analysis for this sample [61].

**Figure 1:**
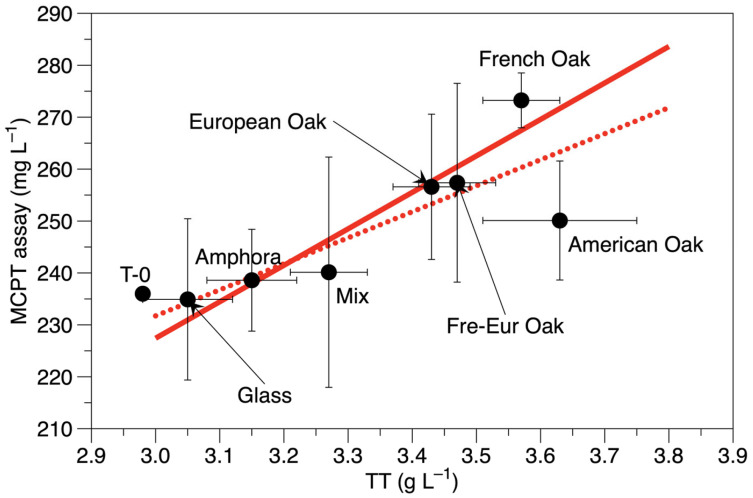
Linear regressions of TT (total tannins) concentration (g L^−1^) and MCPT assay results (mg L^−1^).

As expected, wine in bottles showed the lowest level of total phenols and total tannins compared to the starting wine. On the other hand, the amphora had a significantly higher concentration of total phenols compared to glass bottles. This could imply good protection against the degradation of anthocyanins, as shown below. The MCPT assay is related to condensed tannins and the tannin–polymer interactions [21]. Its values were not affected by different ageing materials, according to Tukey’s HSD test, due to a certain variability of data among replicates. However, Fisher’s LSD test indicated that the MCPT assay values of French oak were higher than those found in bottles, amphora and mixed barrels. The MCPT assay and total tannins showed a linear relation (adjusted R^2^ = 0.578, *p*-value = 0.029; see Supplementary Materials). The linearity consistently increased, excluding the data from American oak (adjusted R^2^ = 0.887, *p*-value = 0.003, see Figure 1), which presented an unexpectedly lower MCPT assay value compared to the observed total tannins content. Therefore, American oak could have released tannins with lower reactivity towards polymers.

Free anthocyanins diminished after ageing in all the samples, as a result of condensation as well as degradation phenomena [62]. The decrease was less intense in European oak, French and European oak and in amphorae. Also, Wang et al. [63] found a higher concentration of anthocyanins in wine aged in unglazed pottery vessels compared to wines aged in other vessels, emphasizing a good behavior of amphorae in protecting anthocyanins. Concurrent with the decrease in anthocyanins, an increase in anthocyanin–tannin complexes was observed in all the samples. French oak and American oak showed the highest condensation activity. Consequently, the ratio between free and complexed anthocyanins diminished, with American oak holding the lowest value. Therefore, American oak appeared to release tannins with a marked attitude towards color stabilization rather than interaction with macromolecules. These results, together with high values of color density and low hue, confirmed a good performance of American oak in stabilizing color, as demonstrated by Hernandez et al. [64]. Additionally, the low hue and high color intensity in this sample reflect favorable color evolution, as they are indices of color stabilization [65]. Interestingly, the anthocyanin–tannin complex concentration also increased in amphora, still emphasizing a good capacity of earthenware vessels to stabilize color [3]. This attitude was also confirmed by the color indices [21]; the high color density value and low hue value indicated an improvement in the chromatic characteristics of wine [66]. This behavior is possibly due to the micro-oxygenation phenomena that occurs throughout earthenware vessel walls, as well as through the traditional lime sealing, even in glazed terracotta, promoting the formation of anthocyanin–tannin complexes [63,67,68].

### 3.2. Volatile Compounds

**Figure 2:**
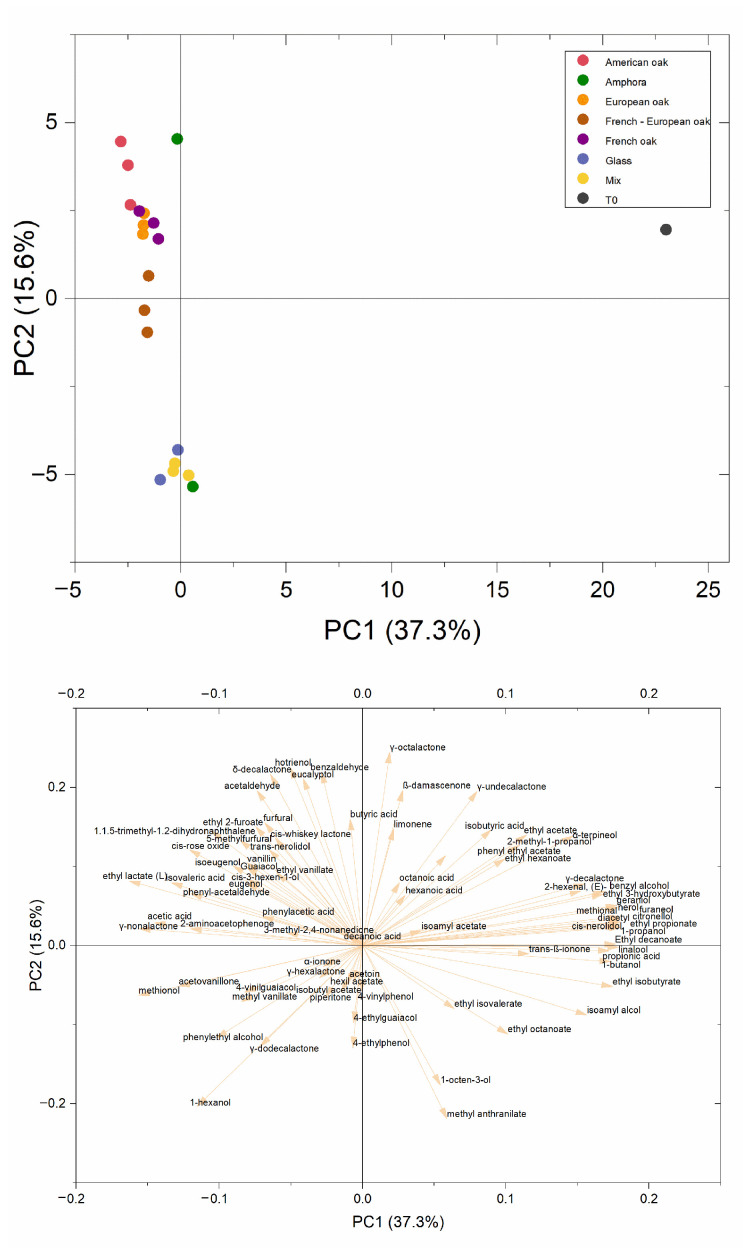
Principal component analysis based on OAVs of volatile compounds found in Negroamaro wines before ageing (T-0) and after ageing in different materials (glass, amphora, mixed, French oak, European oak, American oak, Fre-Eur oak).

The evolution of the volatile pattern after ageing was analyzed by the PCA reported in Figure 2. PC1 explained 37.3% of the variability between the samples. Along this dimension, differences were mainly due to the ageing time, as T-0 was clearly separated from the other samples. As expected, T-0 was characterized by primary aromas, especially several terpenes (geraniol, nerol, linalool, cis-nerolidol), together with fermentation products, such as ethyl esters and acetate esters. At time 0, wine was also characterized by higher alcohols and lactones, such as δ-undecalactone, γ-octalactone and γ-decalactone, which may confer coconutty or fruity/sweet aromas [69]. Acids, which generally represent 10% of Negroamaro’s volatile fraction [15,30], were also abundant. Among acids, the most representative were short-chain fatty acid such as propionic and isobutyric acids, followed by medium-chain fatty acids (hexanoic acid and octanoic acids). After ageing, the wines’ volatile profile significantly changed, as a result of both wine oxidative evolution (e.g., acetaldehyde, benzaldehyde) [70] and esterification phenomena (e.g., ethyl lactate), together with the emergence of woody aromas released from the barrels staves, such as vanillin, eugenol, isoeugenol, furan compounds, etc. [71]. Along PC2 (15.6% variability), samples are distinguished according to the ageing vessels. Interestingly, wines aged in mixed barrels were grouped together with wines in glass bottles, while the others were also grouped together, even though they spread along the axis. Amphorae, which behaved differently along this principal component, were each grouped in a different group. This highlights the expected heterogeneity of handmade amphorae [72]. The PC2, therefore, highlighted a slight shift (15.6% data variability) in the volatile pattern towards the lower-left quadrant, with a decreasing impact of wood and oxidation volatiles and a relative increase in the role of esters, alcohols and volatile phenols. This trend characterized glass and one of the amphorae, as expected, as well as wines aged in French oak barrels, which seemed to provide better modulation of the oxidative evolution of the volatile pattern.

#### 3.2.1. Discrimination Among Woods

**Figure 3:**
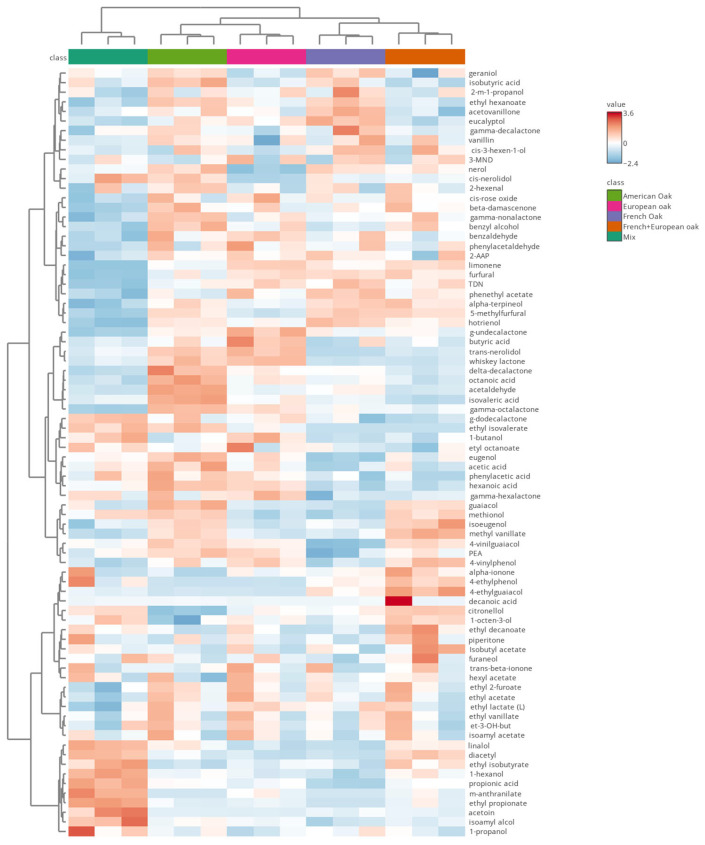
Heatmap with clusterization of the volatile compounds in Negroamaro wine aged in different wooden barrels. Abbreviations for volatile names: 2-m-1-propanol (2-methyl-1-propanol); PEA (phenylethyl alcohol); 3-MND (3-methyl-2,4-nonanedione); 2-AAP (2-aminoacetophenone); ethyl 3-hydroxybutyrate (et-3-OH-but); m-anthranilate (methyl anthranilate); TDN (1,1,5-trimethyl-1,2-dihydronaphthalene).

After six months of ageing, differences among the volatile aroma profiles of the samples aged in different barrels emerged, as in Pérez-Coello et al. [73], demonstrating that, during the first months of ageing, the extraction of aromas from wood to wine reached a maximum. In order to underline these differences, a further elaboration of volatile data found in wood-aged Negroamaro was carried out. Figure 3 presents the heatmap with clusterization of wines and volatiles. The heatmap is based on 80 volatile compounds found throughout gas chromatography, with samples clustered into five different groups, corresponding to the different types of barrels. This confirms the consistency and repeatability of the impact of the type of barrel on the volatile profile of Negroamaro wine [1,73].

Wine aged in mixed barrels demonstrated a quite different volatile profile with respect to the other types of barrels. The mixed sample was characterized by a cluster of volatiles that, in addition to fermentation products (alcohols, acids and some esters), included higher levels of methyl-anthranilate, typical of a foxy character, linalool, which has flowery notes, as previously reported in Negroamaro wines [15] and acetoin and diacetyl, which may result from lactic acid bacteria activities [74]. Moreover, γ-dodecalactone, a lactone typically found in grapes and generally associated with pleasant odors [75], and esters such as ethyl isovalerate and ethyl octanoate were found to be characteristic compounds for wine aged in mixed barrels. American oak was characterized by a cluster of grape lactones including γ-octalactone, δ-decalactone and whiskey lactone; the last one derives from wood and is involved in modifying the perception of fruity aromas, with sweet, peach and coconut notes [44], decreasing red fruits notes and increasing blackberry fruits and spicy scents [76]. The literature reports a higher concentration of oak lactones in American oak compared to European oak [73]. Several volatile phenols were associated with American oak, which may be the result of lignin degradation [77]. Among them, isoeugenol and guaiacol may be related to a positive influence on aroma profile (spicy, vanilla, woody notes), while 4-vinylguaiacol, if present in a high concentration, may confer unpleasant notes [78], although in this sample it was present with very low OAV (0.00128) (data shown in Supplementary Table S2). Figure 3 highlights more intense oxidation processes occurring with American oak, as this sample was characterized by acetaldehyde, the major oxidation by-product of the Fenton reaction [79], phenyl-acetaldehyde, whose correlation with aged and oxidated wines was confirmed by several studies [80,81] and, lastly, acetic acid. Samples aged in European oak were clustered as the most similar to American oak, though with less intense signals for lactone and wood-derived compounds. European oak was marked by compounds such as limonene and *trans*-nerolidol, which are highly characterizing, together with eucalyptol. The Negroamaro wine aged in French oak barrels showed, instead, low levels of volatile phenols and lactones compared to the other types of barrels. French oak profile in the heatmap included two main clusters. The first one comprises two terpenes (α-terpineol and hotrienol), TDN, phenyl ethyl acetate, furfural and 5-methylfurfural. Furfuryl aldehydes are important compounds in oak-aged wines [82]; they impart almonds and toasted almond notes and their presence is found in higher concentrations in French oak compared to American oak [83]. The other cluster includes, besides fermentation products, terpenes (geraniol, eucalyptol), a ketone (acetovanillone), an aldehyde (vanillin) and a lactone (γ-decalactone). Vanillin and its derivatives are wood compounds that confer notes of vanilla, typically reported in wood aged wines [83] and also confirmed in the present study by sensorial analysis. Finally, French + European oak increased the relative levels of volatile phenols and guaiacols. French + European oak was characterized from a group of aromas comprising piperitone, furaneol, citronellol, β-ionone, α-ionone, 4-ethyl phenol and 4-ethyl guaiacol. Some of those are primary aromas associated with positive notes such as piperitone, a terpene reminiscent of mint [84], which may explain the balsamic notes found in this sample during sensory analysis. Furaneol and β-ionone are both associated with red fruit aromas [85]. 4-ethyl phenol and 4-ethyl guaiacol can have a negative influence on wine aroma, and may imply contamination by *Brettanomyces*/*Dekkera* yeasts [77]. Lastly, 1-octen 3-ol resulted as a distinctive compound in French–European oak, and this may derive from vine or grape metabolism and is associated with a mushroomy ‘off’ flavor in wine [86]. In spite of this, the mushroom defect was not reported in this sample during sensory analysis.

**Figure 4:**
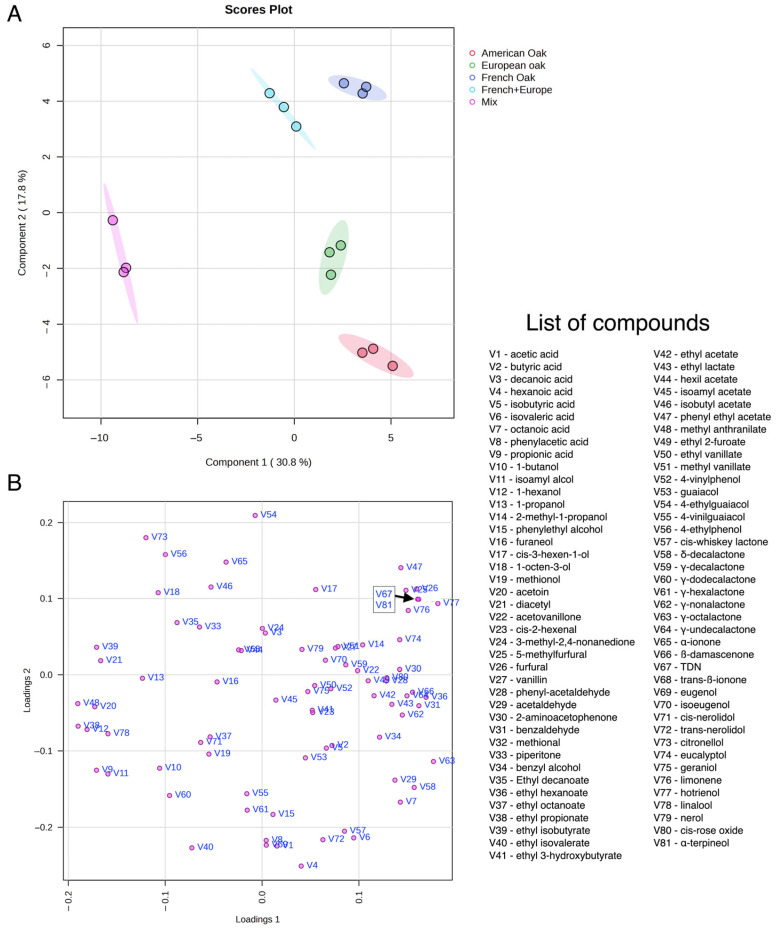
Partial least squares discriminant analysis (PLS-DA) of the volatile compounds in Negroamaro wine aged in different wooden barrels. (**A**) Scores plot with 95% confidence regions. (**B**) Loading plot of the variables. Abbreviations for volatile names: 2-m-1-propanol (2-methyl-1-propanol); PEA (phenylethyl alcohol); 3-MND (3-methyl-2,4-nonanedione); 2-AAP (2-aminoacetophenone); ethyl 3-hydroxybutyrate (et-3-OH-but); m-anthranilate (methyl anthranilate); TDN (1,1,5-trimethyl-1,2-dihydronaphthalene).

**Figure 5:**
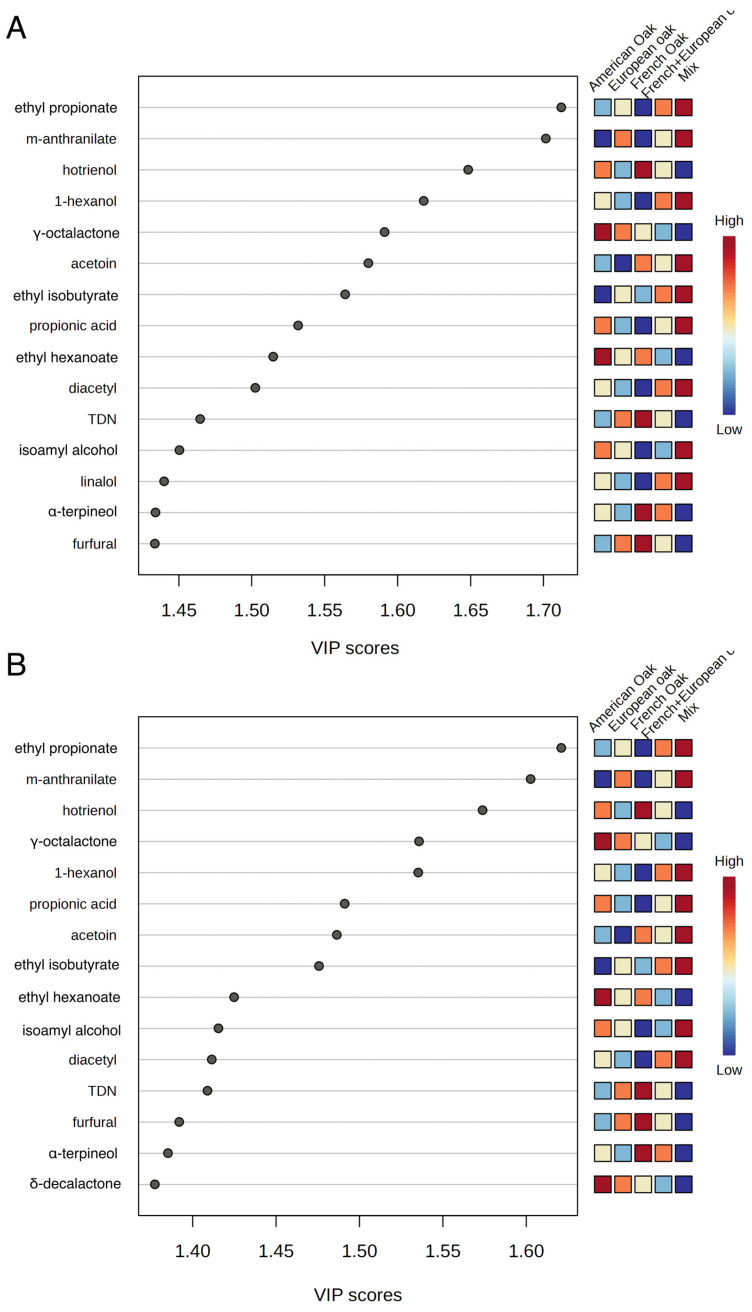
Partial least squares discriminant analysis (PLS-DA) of the volatile compounds in Negroamaro wine aged in different wooden barrels. (**A**) Most important variables in the model according to VIP score for component 1. (**B**) Most important variables in the model according to VIP score for component 2 (the colored boxes on the right indicate the relative concentrations of the corresponding metabolite in each group under study). Abbreviations for volatile names: 2-m-1-propanol (2-methyl-1-propanol); PEA (phenylethyl alcohol); 3-MND (3-methyl-2,4-nonanedione); 2-AAP (2-aminoacetophenone); ethyl 3-hydroxybutyrate (et-3-OH-but); m-anthranilate (methyl anthranilate); TDN (1,1,5-trimethyl-1,2-dihydronaphthalene).

Figure 4 reports the results of the PLS-DA analysis, including the loading plot of the first two components of the model (Figure 4A), the scores plot of the first two principal components with the 95% confidence ellipses (Figure 4B) and the plot of the most important variables in the model according to the weighted sum of absolute regression coefficients (Figure 5A). Samples were clearly discriminated on the plane of the first two components (48% of cumulative variability, Figure 4B), with no overlap of the confidence ellipses.

In terms of PLS-DA, French oak and French + European oak were close but still separated. The similarities are probably due to the presence, in both samples, of French wood. Wine aged in mixed barrels turned out to be the most clearly discriminated. Interestingly, the most important variables involved in the discrimination of wines (Figure 5A,B) are only to a minor extent related to wood-derived compounds. The majority of variables with the highest VIP score, in fact, were esters and varietal aromas, including terpenes (linalool, hotrienol, α-terpineol), norisoprenoids (TDN) and varietal esters (methyl-anthranilate). Therefore, the most relevant difference among the types of barrels on Negroamaro wine ageing was observed in the modulation of varietal and fermentative volatiles, rather than on the release of wood-derived volatiles.

#### 3.2.2. Focus on Some Grape-Derived Compounds

The present section focuses on the fate of some relevant grape-derived compounds after ageing of the Negroamaro wine in vessels of different materials.

##### 3-Methyl-2,4-Nonanedione

**Figure 6:**
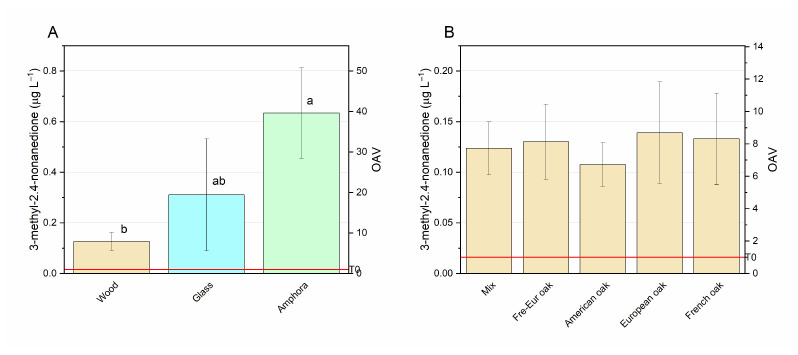
3-methyl-nonanedione concentration in samples aged in wood, glass and amphorae (**A**); 3-methyl-nonanedione concentration in samples aged in different types of barrels (**B**). Different letters mean significant differences at *p* < 0.05. The red line indicates the concentration of the compound before ageing (T0).

In the Negroamaro wines tested in this study, 3-methyl-2,4-nonanedione was present in a higher concentration than its odor threshold (0.016 µg L^−1^). Pons et al. [87] found that its aromatic characteristics depend on its concentration: a range of 0.09–0.17 µg L^−1^ is related to hints of mint and anise; a range of 0.17–0.25 µg L^−1^ confers plum notes; 0.25 µg L^−1^ determines fig nuances; and finally, a value over 0.33 µg L^−1^ determines a rancid odor. At time 0, 3-methyl-2,4-Nonanedione concentration in Negroamaro wine was 0.016 µg L^−1^, while after six months it increased in each sample (Figure 6). This behavior was expected, since 3-methyl-2,4-nonanedione is a β-diketone originating from ketol oxidation during ageing, implying early ageing with the loss of a fresh, fruity and varietal character and the development of dried fruit flavors and cooked fruit nuances [88,89].

Figure 6 discriminates aged samples in different materials in terms of 3-methyl-2,4-nonanedione. Panel A reports the mean values found in wines aged in different materials, and the highest average concentration was observed in wines aged in amphorae. The high values observed in the amphora samples were likely attributable to the oxidative characteristics associated with one of the two amphorae used. This amphora featured a distinct coating that may have resulted in increased gas permeability [72]. In glass samples, as well, concentrations were higher than those found in barrels as the oxygen in the gaseous headspace of the bottle could have been rapidly consumed by oxygen reactive species [90]. Although higher than those of T0, 3-methyl-2,4-nonanedione concentrations in all aged wine samples were in the range related to plum notes and not in the range of unpleasant odors. The only exception was the amphora exhibiting the highest oxidative character, where the concentration (0.467 µg L^−1^, data shown in Supplementary Table S2) could be associated with oxidative odor, according to Peterson et al. [88]. No significant differences were found when comparing the types of barrels (Figure 6B), although all cases exhibited notably higher levels compared to the T-0 wine. This suggests that the formation of 3-MND from its precursors may have occurred during ageing [88], and wood could have led to additional reaction or adsorption of this compound.

However, the levels of 3-MND found in the Negroamaro wine evaluated in the present study were quite high compared to the range reported in the literature [87]. This finding suggests that further research should be carried out on the occurrence and fate of this compound in Negroamaro wines, due to its role in the premature ageing of red wines [87].

##### Furaneol

**Figure 7:**
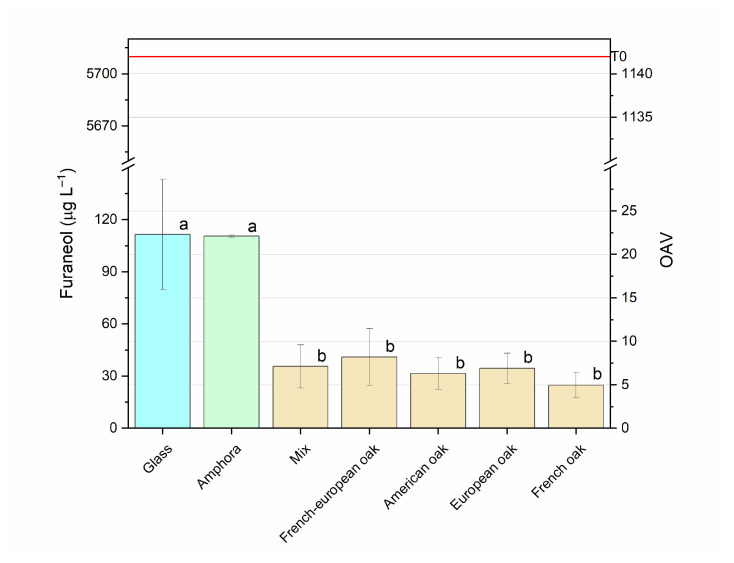
Furaneol concentrations (mean ± S.D.) in Negroamaro wine aged in different materials (glass, amphora, mixed, French oak, European oak, American oak, Fre–Eur oak). Different letters mean significant differences at *p* < 0.05. The red line indicates the concentration of the compound before ageing (T0).

In this study, significant concentrations of furaneol, all notably above its perception threshold (5 μg L^−1^), were found in all samples. Higher concentrations were reached in amphorae and glass, amounting to 110.5 and 111.5, respectively, while barrels accounted for similar values ranging from 24.90 to 41.07 µg L^−1^ (Figure 7). Furaneol is the major furan found in strawberry, generated from sugar degradation with a temperature-dependent process [78]. In some cases, the origin of this molecule in wine has been attributed to wood [91]; in this case, due to the presence of a high concentration even in the absence of wood ageing (glass and amphorae), the first hypothesis can be discarded. This compound therefore has a varietal origin and is found in several Italian red grape varieties; it can enhance the fruity notes of some wines, giving aromas that vary from red fruits (e.g., strawberry) to cooked fruit and intense caramel, depending on its concentration [85,92]. Ageing in amphorae could, therefore, be considered a tool to preserve such varietal compounds in aged wine.

##### β-Damascenone

**Figure 8:**
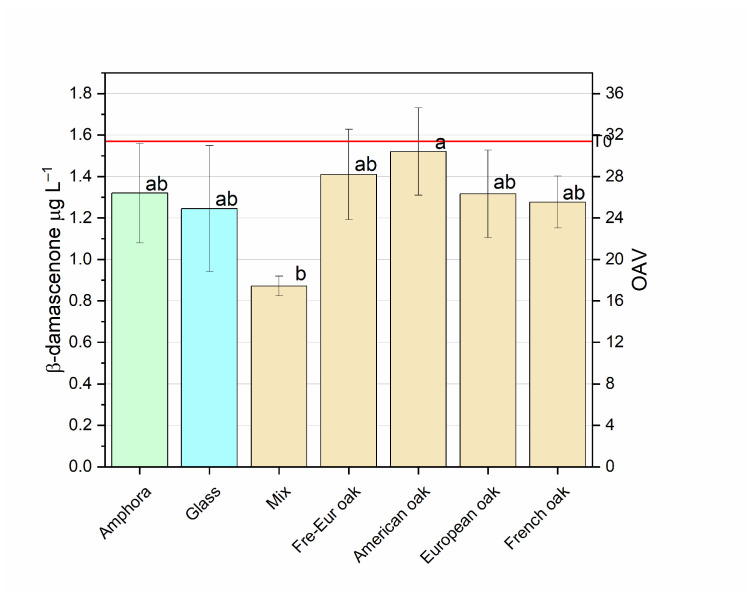
β-damascenone concentrations (mean ± S.D.) in Negroamaro wine aged in different materials (glass, amphora, mixed, French oak, European oak, American oak, Fre-Eur oak). Different letters mean significant differences at *p* < 0.05. The red line indicates the concentration of the compound before ageing (T0).

β-damascenone is a C-13 norisoprenoid carotenoid-derived aroma compound. This ketone can be associated with honey-like, flowery aromas and is considered to enhance fruity aromas [93]. Its presence is due to the degradation of neoxanthine carotenoid compound or to glycosylated precursors through the acid-catalyzed conversion of megastigma-6,7-diene-3,5,9-triol and megastigma-5-ene-7-yne-3,9-diol-derived compound of lutein [94]. Concentrations of β-damascenone in red wines range between 0.5 and 4 µg L^−1^ with a threshold of 0.05 µg L^−1^ in model wines [28]. Different studies have shown some characteristics of β-damascenone, and it has been observed that in red wines it enhances some fruity aromas, hiding the herbaceous aroma of isobutyl methoxypyrazine, which could suggest an indirect sensorial impact rather than a direct effect on red wines’ sensorial bouquet [45]; in young white wines it showed a negligible effect on aroma [94]. Figure 8 reports β-damascenone concentrations in the Negroamaro wine before and after ageing in different types of vessels. In all cases, 6 months of ageing reduced its concentrations in wines. This trend has been confirmed in the literature [95], where a decrease was observed at the end of the ageing (both in models and real wines). This could be due to the diverse precursors of grape varieties and to the acid-catalyzed reaction degrading the C13-norisoprenoid itself. Specifically, the amount of β-damascenone decreased with the greatest reduction, by 44%, in mixed wood barrel samples, while, on the other hand, the American oak sample was pretty similar to the control, with a reduction by 3%. Glass and amphorae showed a similar amount, with a 21% and 16% decrease, respectively. One-way ANOVA showed significant differences between American oak and mixed wood barrel samples (*p* < 0.05).

##### TDN

**Figure 9:**
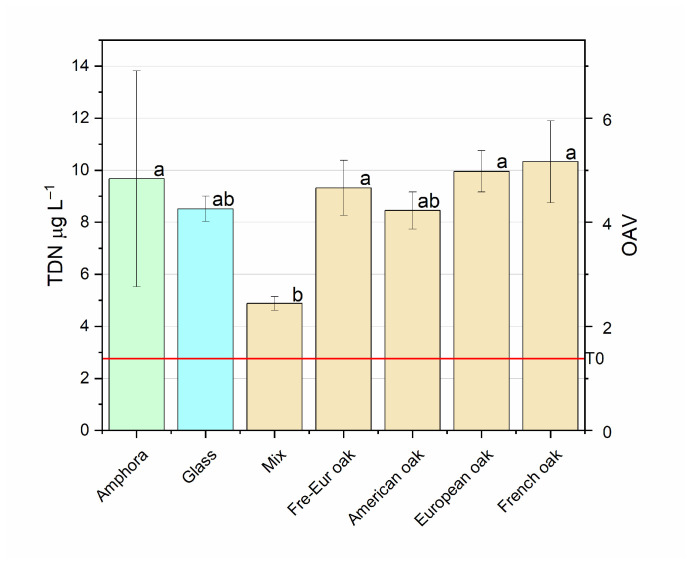
TDN (1,1,5-trimethyl-1,2-dihydronaphthalene) concentrations (mean ± S.D.) in Negroamaro wine aged in different materials (glass, amphora, mixed, French oak, European oak, American oak, Fre-Eur oak). Different letters mean significant differences at *p* < 0.05. The red line indicates the concentration of the compound before ageing (T0).

TDN (1,1,5-trimethyl-1,2-dihydronaphthalene) is a C13-Norisopenoid compound which typically characterizes aged wines (particularly aged Riesling wines) [46]. If present in high amounts, it typically confers kerosene or petrol-like aromas, masking other sensorial compounds, which make it unsuitable for consumers [46]. The most common concentration in European wines ranges between 5 and 50 μg L^−1^, but it is not unusual to find 250 μg L^−1^ in Australian wines [96]. TDN is found at low levels in grapes and young wines, while its levels increase during wine ageing as the result of the progressive hydrolysis of glycosidic odorless precursors [46]. However, increasing levels have been reported in the last few decades and have been related to climate change [46]. Its content in grapes is generally low, although different viticulture techniques, such as defoliation (direct effect) or water irrigation (undirect effect), can affect the formation of TDN in wines [97]. The perception threshold is constantly under study due to differences in its sensory impact; it is currently reported at 2 µg L^−1^ [46]. Figure 9 reports the amount of TDN after ageing in different vessels, compared with the level found before ageing. In all cases, TDN increased in all samples, with the highest peak in French oak, increasing by 274%, and the lowest peak in mixed, increasing by 77%. All other samples were similar, with an increasing concentration in the range of 206–261%. This trend confirms previously mentioned works in which wine ageing was reported to increase the concentration of TDN. The comparison between 6-month-aged wines showed no differences among French oak, European oak, French–European wood barrel samples and amphorae samples, while the mixed sample showed significant differences compared to all the other samples. Glass and American oak wood barrels showed central values.

### 3.3. Sensory Properties

**Figure 10:**
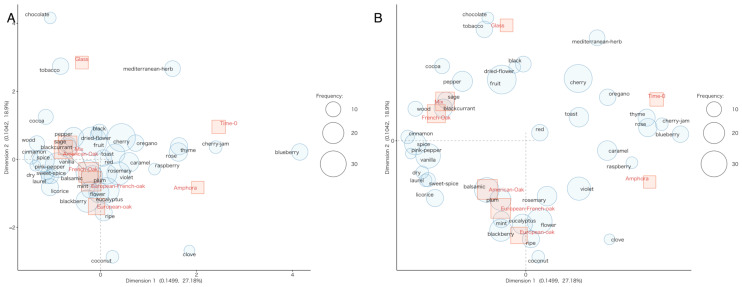
Correspondence analysis of the aroma attributes of the Negroamaro wine before ageing (T-0) and after ageing in different materials (glass, amphora, mixed, French oak, European oak, American oak, Fre-Eur oak). (**A**) Original scale map; (**B**) map with the “enlarge the area near origin” function (3×). Red squares correspond to wines and blue bubbles correspond to taste attributes. Size of bubbles represents term frequency.

Figure 10 reports the sensory space of samples derived from the results of the correspondence analysis carried out on the frequency of occurrence of free-choice olfactory descriptors of wines at time 0 and after ageing in different containers. Samples were grouped considering the frequency of attribution of aroma descriptors to the samples. The panel on the right side highlights the differences among aged wines when applying the function of magnification of the area near the origin included in the analysis software. A clear distinction emerged among samples: time 0 was different from all aged wines, with notes of mediterranean herbs, oregano and thyme that are typically reported in Negroamaro wines [98,99], as well as some floral (rose) and fruity notes (cherry, blueberry, cherry jam). After six months of ageing, the wines’ aroma was enriched and samples were distinguished according to the container used, with wood origin determining a differentiation as highlighted in Figure 10B. In particular, ageing in amphorae imparted distinct olfactive characteristics, with a slight evolution of the fruity and floral notes, associated with raspberry and violet, and descriptors including caramel and clove. Hence, amphorae determined a varietal aroma evolution without covering it with woody notes, in agreement with previous reports [3]. This profile was quite different from that deriving from bottle ageing, characterized by increased tobacco and chocolate notes. A slight differentiation in olfactory profiles could be observed among different woods. Barrels containing European and American oak imparted notes ranging from balsamic (together with mint, eucalyptus and rosemary) to coconut and plum. This could be related to higher levels of lactones [44]. On the other hand, French oak and mixed barrels kept fruity notes (such as black fruits and blackcurrant) and imparted spicy characteristics.

**Figure 11:**
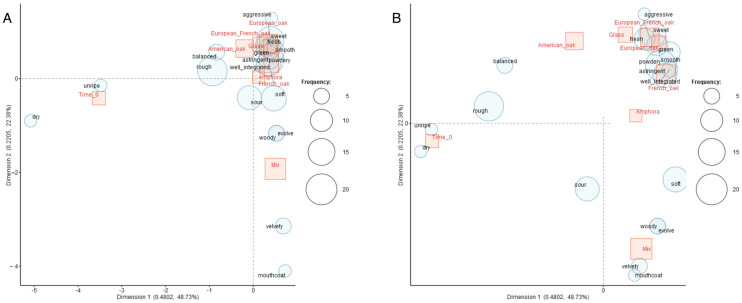
Correspondence analysis of the taste attributes of the Negroamaro wine before ageing (T-0) and after ageing in different materials (glass, amphora, mixed, French oak, European oak, American oak, Fre-Eur oak). (**A**) Original scale map; (**B**) map with the “enlarge the area near origin” function (3×). Red squares correspond to wines and blue bubbles correspond to odor attributes. Size of bubbles represents term frequency.

Figure 11 reports the sensory space of samples deriving from the results of the correspondence analysis carried out on the frequency of occurrence of free-choice taste descriptors of wines at time 0 and after ageing in different containers. The map clearly shows the effect of ageing on the Negroamaro astringency. At time 0, the wine was described as unripe, dry and rough, a description related to negative hedonic properties [100], while six months of ageing determined an evolution of mouthfeel as wine acquired a more complex profile, though panelists still found some aggressive traits in the wine, such as astringency, after six months of ageing. Only wine aged in the mixed barrels clearly showed an interesting evolution of the astringency, with velvety, mouthcoat and evolved taste descriptors noted. This kind of barrel therefore provided the best performances in a six-month ageing period on Negroamaro wine.

## 4. Conclusions

The present study pointed out several differences determined by different ageing packaging materials on Negroamaro wine, producing wines with unique chemical and sensory characteristics (Table 3). Wood barrels, particularly of French and American oak, significantly increased total phenolic compounds and stabilized anthocyanin–tannin complexes, enhancing color intensity and complexity. Amphorae effectively preserved phenolic content and color stability. A clear differentiation could be observed in the evolution of the volatile profile with regard to volatiles released, as well as the pattern of varietal aromas. As a consequence, the sensory profile of the Negroamaro wine could be led to different outcomes. Amphorae kept primary fruity and floral aromas with a specific ageing evolution. Wooden barrels contributed distinct aromatic profiles based on wood type. The findings reveal significant differences in the influence of ageing containers and highlight the promising potential of traditional Apulian amphorae as a sustainable and versatile ageing container in winemaking. Future research should focus on optimizing amphora size and coatings to ensure consistency and explore their long-term impact on wine quality. The revival of these traditional vessels could contribute to sustainable winemaking practices and the valorization of regional heritage, offering a unique identity to wines from Apulia.

## Figures and Tables

**Table 1:** Characteristics of different types of barrels used for ageing (TN Coopers, 2021–2022).

Name of the Barrel	Characteristics	
Mixed	Blend of French oak (*Q. petraea* (Matt.) Liebl.), American oak (*Q. alba* L.), European oak (*Q. petraea* (Matt.) Liebl.), acacia (*Robinia pseudoacacia* L.) and lenga (*Nothofagus pumilio* (Poepp. & Endl.) Krasser)	Dried up to 48 months; fine/extra fine grain
American oak	*Q. alba* L.	Air-dried up to 48 months; fine grain; mature wood over 90 years old
French oak	*Q. petraea* (Matt.) Liebl.	PEFC certification; dried up to 36 months; fine grain; mature wood 180 years old; cultivated with the Haute Futaie technique (tall trunk)
European oak	*Q. petraea* (Matt.) Liebl.	PEFC certification; fine grain; air-dried up to 48 months
French–European oak	Blend of French oak (*Q. petraea* (Matt.) Liebl.), European oak (*Q. petraea Q. petraea* (Matt.) Liebl.)	Dried up to 48 months; fine/extra fine grain

**Table 2:** TP (total phenols) (absorbance units, a.u.), TA (total anthocyanins) (absorbance units, a.u.), TT (total tannins), FA (free anthocyanins), A-T (anthocyanin–tannin complex), free/cond A (free and condensed anthocyanins ratio), MCPT (methyl cellulose precipitable tannins), CD (color density) and hue of the wine at time 0 (T-0) and of the wines obtained using different ageing materials. Results of multiple comparisons among aged wines after one-way ANOVA are reported *.

	T-0	*p*	Glass	Amphora	Mixed	Fre–Eur Oak	American Oak	European Oak	French Oak
TP (a.u.)	79.77	0.00	79.25 ± 0.21 ^e^	81.3 ± 0.28 ^cd^	80.83 ± 0.60 ^d^	84.3 ± 0.46 ^b^	82.13 ± 0.06 ^c^	84.7 ± 0.20 ^b^	86.3 ± 0.00 ^a^
TA (a.u.)	20.1	0.00	16.3 ± 0.00 ^d^	18.35 ± 0.07 ^b^	17.53 ± 0.06 ^c^	18.4 ± 0.17 ^b^	16.03 ± 0.15 ^d^	18.9 ± 0.10 ^a^	17.67 ± 0.15 ^c^
TT (g L^−1^)	2.98	0.00	3.05 ± 0.07 ^c^	3.15 ± 0.07 ^c^	3.27 ± 0.06 ^bc^	3.43 ± 0.06 ^ab^	3.63 ± 0.12 ^a^	3.47 ± 0.06 ^ab^	3.57 ± 0.06 ^a^
FA (U)	13.5	0.00	9.2 ± 0.00 ^ab^	9.65 ± 1.20 ^ab^	9.1 ± 0.1 ^b^	10.13 ± 0.06 ^a^	5.43 ± 0.06 ^c^	10.23 ± 0.12 ^a^	8.67 ± 0.06 ^b^
A-T (U)	3.8	0.03	4.1 ± 0.00 ^c^	5.1 ± 0.71 ^b^	4.87 ± 0.06 ^b^	4.87 ± 0.06 ^b^	6.33 ± 0.06 ^a^	4.93 ± 0.06 ^b^	5.33 ± 0.06 ^b^
free/cond A	3.6	0.00	2.24 ± 0.00 ^a^	1.93 ± 0.50 ^ab^	1.87 ± 0.04 ^ab^	2.08 ± 0.04 ^a^	0.86 ± 0.00 ^c^	2.07 ± 0.02 ^a^	1.63 ± 0.02 ^b^
MCPT (mg L^−1^)	236	0.04	234.91 ± 15.54 ^a^	238.60 ± 9.81 ^a^	240.14 ± 22.18 ^a^	256.59 ± 1.23 ^a^	250.11 ± 11.46 ^a^	257.37 ± 19.14 ^a^	273.24 ± 5.28 ^a^
CD	11.12	0.01	10.91 ± 0.19 ^b^	13.00 ± 1.62 ^a^	12.91 ± 0.22 ^a^	12.86 ± 0.31 ^ab^	13.82 ± 0.42 ^a^	12.67 ± 0.37 ^a^	12.89 ± 0.41 ^a^
Hue	0.84	0.00	0.86 ± 0.01 ^a^	0.80 ± 0.04 ^b^	0.80 ± 0.00 ^b^	0.79 ± 0.01 ^b^	0.76 ± 0.03 ^b^	0.80 ± 0.00 ^b^	0.79 ± 0.01 ^b^

*, Tukey’s HSD test for multiple comparisons. Different letters indicate significantly different means (*p* < 0.05).

**Table 3:** Summary table of the most significant differences in phenolic indices, volatile compounds and sensorial profile of wines aged in wood, amphora and glass.

	Woods	Amphora	Glass
Phenolic indices	French oak had the highest total phenols and tannins. European and French–European oak minimized anthocyanin degradation, while American oak excelled in color stabilization with high condensation activity and a low free/complexed anthocyanin ratio.	Good protection against anthocyanin degradation. High anthocyanin–tannin condensation activity, related to an improvement in chromatic stability. High color density and low hue.	Lowest total phenol and total tannin concentrations, comparable to T0. Low color density, high hue.
Volatile compounds	Distinct markers for each wood type. American oak was characterized by lactones and oxidation compounds, the mixed type had a unique profile with respect to other woods and the highest β-damascenone reduction and French oak was rich in vanillin and its derivatives.	Preserved fruity and floral volatiles (e.g., furaneol). Higher concentration of 3-methyl-2,4-nonanedione. Preserved terpenes (geraniol, linalool) and other varietal compounds. Slight oxidation.	Minimal presence of oxidation compounds. Retention of higher alcohols and acids. Preserved primary aromas (e.g., terpenes, esters). High concentration of Furaneol.
Sensorial profile	Evolved with wood influences, varying by barrel type. European and America oak contributed balsamic notes, coconut and plum. French oak and mixed barrels imparted vanilla and spicy notes. The mixed type improved astringency, with the result described as velvety and mouthcoating.	Evolution without overshadowing primary characteristics. Preservation of varietal fruity and floral aromas (raspberry, violet). Introduction of caramel and clove nuances.	Development of tobacco and chocolate notes. Less aromatic complexity compared to other ageing materials.

## Data Availability

The original data presented in the study are openly available in FigShare at https://doi.org/10.6084/m9.figshare.28182521.

## Supplementary Materials

The following supporting information can be downloaded at: https://www.mdpi.com/article/10.3390/foods14040650/s1, Figure S1: One of the amphorae (ozza) used in the experimental trial. Figure S2: Sample chromatogram of the SPME-GC/MS analysis of volatile compounds. Table S1. Retention times of the analysed volatile compounds. Table S2. Volatile compounds concentrations and odour activity values (OAV) at time 0 and after aging in vessels of different materials. Mean a values and standard deviations (n = 3) of experimental replicates are reported. One-way ANOVA and Tukey’s HSD test were applied on aged wines (different letters mean a significant difference at *p* < 0.05). References for odour tresholds are reported in a dedicated column.
