# Supplementary material for: Ageing of Red Wine (cv. Negroamaro) in Mediterranean Areas: Impact of Different Barrels and Apulian Traditional Amphorae on Phenolic Indices, Volatile Composition and Sensory Analysis
Source: Foods. 2025 Feb 14;14(4):650. doi: 10.3390/foods14040650 (PMC11854446; doi:10.3390/foods14040650)
Supplement: Supplementary file 1 [file foods-14-00650-s001.zip › Prezioso et al - Supplementary Figures.pdf]

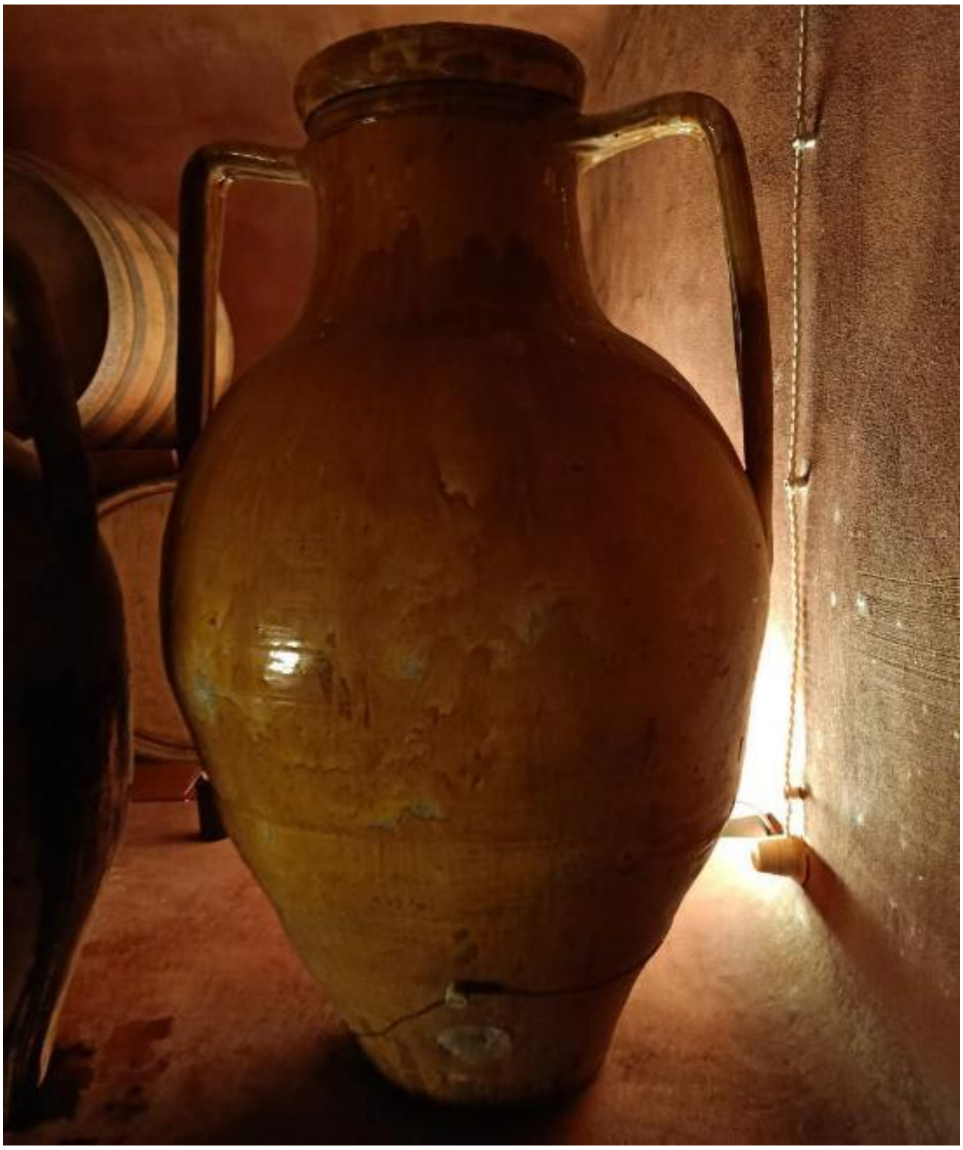

Figure S1. One of the amphorae (*ozza*) used in the experimental trial.

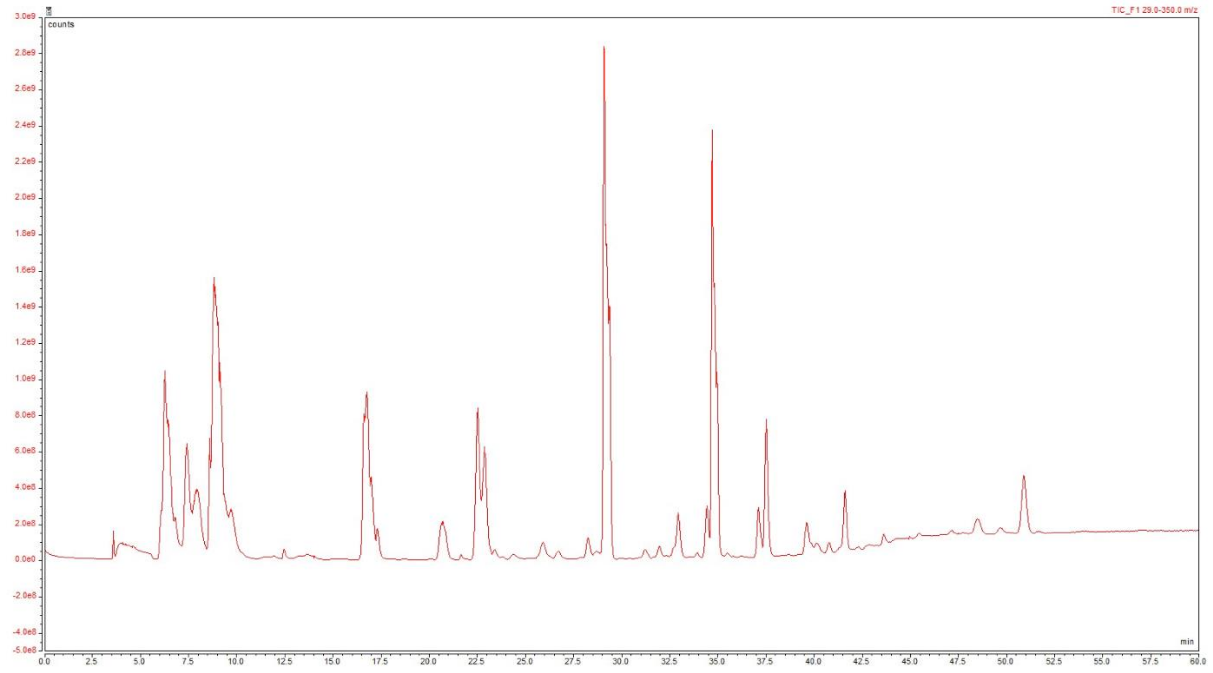


Figure S2. Sample chromatogram of the SPME-GC/MS analysis of volatile compounds.
